# HLA class I expression and tumor immune infiltration together shape colon cancer immune contexture

**DOI:** 10.1016/j.neo.2026.101357

**Published:** 2026-09-02

**Authors:** Hehuan Zhu, Roos van Nieuwkerk, Savannah van der Kroft, Ronald L.P. van Vlierberghe, Marieke E. IJsselsteijn, Weimin Zuo, Thomas Vauvert F. Hviid, Rob A.E.M. Tollenaar, Alexander L. Vahrmeijer, Davide Bedognetti, Wouter R.L. Hendrickx, Jessica Roelands, Peter J.K. Kuppen

**Affiliations:** a Department of Surgery, Leiden University Medical Center, Leiden, Netherlands; b Department of Pathology, Leiden University Medical Center, Leiden, Netherlands; c Guangdong Provincial Key Laboratory of Autophagy and Major Chronic Non-Communicable Diseases, Affiliated Hospital of Guangdong Medical University, Zhanjiang, China; d Department of Clinical Biochemistry, Centre for Immune Regulation and Reproductive Immunology (CIRRI), Zealand University Hospital, Copenhagen, Denmark; e Department of Clinical Medicine, University of Copenhagen, Copenhagen, Denmark; f The ReproHealth Research Consortium, Zealand University Hospital, Copenhagen, Denmark; g Tumor Biology and Immunology Lab, Research Branch, Sidra Medicine, Doha, Qatar

**Keywords:** Colon cancer, HLA class I, Antigen presentation, Immune infiltration, Immune contexture

## Abstract

**Background:**

The relative contribution of HLA class I molecules—including classical HLA class Ia (HLA-A, -B, -C) and non-classical HLA class Ib (HLA-E, -F, -G)—to shaping the tumor immune microenvironment in colon cancer remains insufficiently defined. We investigated how their expression patterns relate to immune infiltration and clinical outcome.

**Methods:**

In a retrospective cohort of 280 colon cancers, we assessed HLA class Ia and class Ib expression and quantified CD45-positive immune cell infiltration by immunohistochemistry. These features were correlated with clinicopathological variables, microsatellite instability (MSI) status, and previously established genomic immune signatures.

**Results:**

High HLA class Ia expression and increased CD45-positive cell infiltration were each associated with improved overall, disease-specific, and progression-free survival. CD45-positive density correlated strongly with Immunologic Constant of Rejection scores. HLA class Ia loss was more frequent in advanced stages and in MSI-H tumors. Among HLA class Ib molecules, only HLA-E expression was associated with favorable disease-specific and progression-free survival. Integrative analysis identified three immune phenotypes with distinct prognostic profiles; tumors characterized by high HLA class Ia expression, low HLA class Ib expression, and high CD45-positive infiltration had the best outcomes.

**Conclusion:**

Colon cancer immunogenicity is shaped by coordinated patterns of HLA class I expression and immune infiltration. Integrating HLA class Ia/Ib expression with immune cell density provides a refined stratification of tumor immune phenotypes and may support personalized immunotherapeutic decision-making.

## Introduction

Colon cancer continues to pose a major challenge to global health and is one of the leading causes of cancer-related deaths worldwide [2]. Despite continuous advances in surgical techniques [3], and other therapeutic options [4,5], patient outcomes remain highly heterogeneous, particularly among patients with intermediate-stage disease [[6], [7], [8]]. The tumor immune microenvironment (TIME) plays a key role in influencing disease progression and shaping therapeutic responses [[9], [10], [11]]. The clinical success of immune checkpoint inhibitors, especially anti-PD-1/PD-L1 therapy in microsatellite instability-high (MSI-H) colon cancer highlights the importance of immune surveillance in colon cancer patients [12,13]. However, the vast majority of tumors—especially microsatellite stable (MSS) cases—remain unresponsive to immunotherapy [14,15], suggesting either insufficient immune priming or active immune evasion as potential barriers to response. Immune infiltration, antigen presentation, and immune evasion mechanisms collectively influence tumor behavior, and thus a comprehensive framework is needed to better understand tumor-immune interactions.

The human leukocyte antigen (HLA) class I family comprises two main groups: class Ia molecules (HLA-A, -B, and -C), which present tumor-derived antigens to CD8⁺ cytotoxic T lymphocytes (CTLs), and class Ib molecules (HLA-E, -F, and -G), which are primarily involved in immune modulation [16,17]. Defects in HLA class Ia expression is a well-documented immune escape strategy that allows tumor cells to evade T cell-mediated cytotoxicity [18,19]. In contrast, HLA class Ib molecules (HLA-E, HLA-F, and HLA-G) affect immune regulation by binding to inhibitory receptors on natural killer (NK) cells and T cells, or other immune cells, further creating a balance between immune activation and immune inhibition [17,[19], [20], [21]]. The relationship between these molecules and the clinical outcome of colon cancer remains incompletely understood.

In addition to antigen presentation, the location, tumor stroma or tumor epithelium, and density of the immune infiltrate has an important impact on tumor progression and response to therapy. CD45 is a pan-leukocyte marker that serves as an indicator of overall immune cell infiltration and is associated with better patient outcomes in a variety of malignancies [[22], [23], [24], [25]]. Previous studies have explored the relationship between antigen presentation and immune cell infiltration in colon cancer, including the role of HLA class Ia in regulating T-cell responses [26], but the specific relationship between the level of HLA class Ia expression and the density of CD45-positive tumor-infiltrating immune cells, especially in relation to DNA microsatellite status and clinical outcome, remains to be fully elucidated. In addition, it is well known that MSI-H tumors have enhanced immunoreactivity due to increased neoantigen load [18,27]. Paradoxically, these tumors often show defects or complete loss of HLA class Ia expression, a phenomenon widely interpreted as a consequence of immune-mediated selective pressure [18,28]. This suggests that despite robust immune activation, MSI-H tumors may escape T cell-mediated killing through impaired antigen presentation.

Given the complexity of tumor immune interactions, assessment of individual immune markers may not be sufficient to reflect their impact on patient prognosis. Instead, a composite immunophenotyping approach integrating multiple immune parameters may provide a more comprehensive and biologically meaningful tumor classification. This builds on our previous work, in which we demonstrated that combined expression patterns of HLA class I molecules (HLA-Ia, HLA-E, and HLA-G) were associated with microsatellite status, immune evasion markers, and patient survival in colon cancer [29]. In this study, we systematically analyzed the tumor expression of HLA class Ia (HLA-A, -B, -C) and HLA class Ib molecules (HLA-E, -F, and -G), as well as the extent of immune cell infiltration, in a cohort of colon cancer patients. Furthermore, we investigated their separate and mutual associations with clinicopathological features, including DNA microsatellite status, Consensus Molecular Subtypes (CMS), Immunologic Constant of Rejection (ICR), and clinical outcome.

Our findings provide further insights into the relationship between the expression of HLA class Ia and class Ib, immune cell density, DNA microsatellite status, and patient survival in a cohort of colon cancer that is extensively studied for genomic and spatial immune parameters [30,1]. By comprehensively analyzing these markers, we identified unique immune signatures that may reflect underlying mechanisms of immune control or evasion. These observations highlight the importance of considering both immune cell infiltration and antigen presentation when characterizing the TIME.

## Methods

### Patient cohorts

From the original AC-ICAM project, formalin-fixed paraffin-embedded (FFPE) primary tumor samples were available from 280 patients. The collected patient information was de-identified and the tissue samples were handled in accordance with the guidelines outlined in the Code of Conduct for Proper Secondary Use of Human Tissue of the Dutch Federation of Biomedical Scientific Societies, ensuring anonymity. The study was conducted in accordance with the principles described in the Helsinki Declaration and was approved by the IRB at LUMC and Sidra Medicine (IRB: 1768087-1, 1602002725 and B19.079). This study is reported in accordance with the STROBE guidelines (cohort).

### Antibodies

The mouse monoclonal antibody EMR8-5 (ab70328, AbCam, Cambridge, UK) was used to detect HLA class Ia molecules (HLA-A, -B, and -C) [31]. A mix of rabbit polyclonal antibodies targeting collagen I, collagen VI, and elastin (ab34710, ab6588, and ab23747 respectively, all from AbCam) was used in order to stain extracellular matrix (ECM), stromal tissue and blood vessels in tumor tissue, to distinguish tumor stroma from tumor epithelium. A mouse monoclonal anti-CD45 antibody (clone 2B11+PD7/26; DAKO, Denmark) was included to visualize tumor-infiltrating immune cells. To detect HLA class Ib molecules, the following monoclonal antibodies were used: HLA-E (clone MEM-E/02; Abcam, UK), HLA-F (clone 3D11; Novusbio, USA), and HLA-G (clone 4H84; Nuclilab, The Netherlands). For each antibody, the dilution to obtain optimal immunohistochemical staining was determined, using proper positive and negative control tissues.

### Immunohistochemistry

Four independent IHC double staining combinations were prepared to evaluate the expression of HLA class Ia, HLA-E, and HLA-G, as well as tumor-infiltrating CD45-positive immune cell density, in relation to the extracellular matrix (ECM)/ tumor stroma [32]. Briefly, 4 μm thick FFPE tissue sections were deparaffinized and rehydrated, followed by heat-induced antigen retrieval in Envision FLEX target retrieval solution low pH (DAKO, Glostrup, Denmark) using a PT Link module (DAKO). Endogenous peroxidase and phosphatase activity was blocked for 10 minutes using BloxAll solution (Vector Laboratories, Burlingame, CA, USA). Each antibody mix contained one mouse primary antibody (EMR8-5, MEM-E/02, 4H84, or 2B11+PD7/26) and a rabbit polyclonal antibody mix against ECM components (collagen I, collagen VI, and elastin), diluted in 1% BSA in PBS. Slides were incubated overnight at room temperature with the corresponding antibody combinations. The next day, chromogenic detection was performed sequentially. For the HLA class Ia–ECM, HLA-E–ECM, and HLA-G–ECM stainings, the rabbit antibodies were developed first using anti-rabbit HRP polymer (Rabbit Envision; DAKO) and DAB substrate (DAKO), followed by the mouse antibody using anti-mouse alkaline phosphatase polymer (MACH-2; Biocare Medical) and development with Vector Blue (Vector Laboratories). For the CD45–ECM staining, the detection order was reversed: CD45 was developed first with anti-mouse HRP polymer and DAB, followed by ECM detection with anti-rabbit AP polymer and Vector Blue. Note that the tissue sections were not counterstained with hematoxylin, like in a standard IHC staining procedure. Finally, the sections were washed in demineralized water and mounted with Ecomount (Biocare Medical). Due to technical constraints, HLA-F expression was assessed using conventional single IHC staining, following the protocol like described in our previous study [17].

### Evaluation of immunohistochemistry

The procedure for the semi-automated image analysis regarding HLA class Ia IHC double staining has been previously described [33]. Microscopic analysis of HLA-E and HLA-F was independently evaluated in a blinded manner by two observers (R.v.N., H.Z.). Similarly, HLA-G analysis was assessed by two independent observers (S.v.d.K., H.Z.), as well as CD45 analysis was conducted by two independent observers (R.L.P.v.V., H.Z.), all under blinded manners. A Cohen's Kappa value above 0.8 was predefined as the threshold for acceptable interobserver concordance, based on established interpretation guidelines [34]. The Cohen's Kappa was >0.8 for all quantifications, indicating substantial agreement between the two observers. For HLA class Ia, the expression was assessed based on the percentage of positive staining area (pixel-based analysis), and the expression status was determined according to the standard set by the International HLA and Immunogenetics Workshop [35]. To ensure staining quality and interpretation reliability, only slides with detectable internal positive control (e.g., stromal immune cells, endothelial cells, or normal epithelial glands) were included for further analysis. HLA class Ia expression levels were categorized into three groups based on the percentage of positive staining tumor epithelial area: 0–25%, 25–75%, and 75–100%. Tumors with <25% positive area were classified as HLA class Ia Negative. Those with 25–<75% were categorized as having Low HLA class Ia expression, and ≥75% as High HLA class Ia expression. For subsequent analyses, tumors with ≥25% expression (i.e., the Low and High groups) were combined and referred to as the Retained group; tumors with <25% expression were referred to as the Loss group. For HLA-E or HLA-G, the expression was categorized by the percentage of positive staining tumor epithelial area into three groups: 0-25% (Negative), 25-75% (Low), and ≥75% (High). For subsequent analyses, Low and High were combined as the Positive group, versus the Negative group. For HLA-F, fixed rectangular regions of interest (ROIs) of 1.0 mm² were placed within tumor areas (N = 3 per case) on whole-slide images. Only tumor cells were counted. A positive cell was defined as cytoplasmic or membrane staining of any intensity. Cases were classified as Negative (≤10 positive cells/ROI), Low (11-19/ROI), or High (≥20/ROI). For subsequent analyses, Low and High were grouped as HLA-F Positive, compared to the Negative group. For CD45, we quantified the density of CD45-positive cells infiltrating the tumor epithelium manually on whole-slide scanned sections. For each slide, four ROIs were randomly selected within the tumor epithelium, and CD45-positive cells were counted within each ROI and normalized to the area (expressed as cells/mm²). Then all samples were categorized into CD45 high-density and low-density groups based on the median value.

### Transcriptome and whole exome sequencing data

CMS classification, ICR clusters, and MSI status based on MANTIS [36] were previously determined [1]. Data was available in the Supplementary Source Data File corresponding to the original publication (Gene expression matrix in Supplementary Source Data 3, CMS classification in Supplementary Source Data 4, ICR clusters in Supplementary Source Data 19, and MSI in Supplementary Source Data 11).

### Statistical analysis

All statistical analyses were performed using the IBM SPSS (version 25; Armonk, NY) and GraphPad Prism software (version 8.0d; GraphPad, San Diego, CA, USA) unless stated otherwise. Statistical analyses of quantification were performed with the two-tailed Mann–Whitney U-test between two groups and the Kruskal–Wallis test among multiple groups as appropriate. For survival analyses, Kaplan–Meier plots were drawn, and statistical differences were evaluated using the log-rank Mantel–Cox test. Furthermore, the expression of HLA class Ia and density of CD45-positive cells were correlated with next generation sequencing data using Pearson correlation tests. Univariate and multivariate prognostic analyses were done using Cox regression analysis (Wald test). The variables tested in the univariate analysis included patients’ age and sex, anatomical location of the tumor, adjuvant treatment, AJCC stage, MSI status, ICR-based classification, CMS classification, HLA class Ia expression, CD45-positive cell density and the immune phenotypes. Multivariate analysis incorporated all variables with a *p*-value lower than 0.05 in univariate analysis. Cox proportional hazard models were built to investigate whether the immune phenotypes improved the prognostic value of T cell related immune signatures, such as ICR classification. A *p*-value of ≤ 0.05 was considered statistically significant.

## Results

### Expression of HLA class Ia and class Ib (HLA-E, -F, and -G) and CD45-positive cell density

All available formalin-fixed, paraffin-embedded (FFPE) samples from a well-characterized, previously established multi-omics cohort of colon cancer patients [30,1] were collected. The resulting 280-patient cohort included a representative mix of colon tumors from stage I to IV, with the majority diagnosed at stages II or III. Tumors were approximately evenly distributed between the left and right colon sides, and clinical characteristics such as age, gender, and treatment history reflected a representative population of colon cancer patients (**Table S1**).

A summary for this study population, sample and data availability, including IHC-evaluable cases (HLA-Ia, HLA-E, HLA-F, HLA-G 272 and CD45), endpoint availability, and the intersections used for composite phenotypes, was provided (**Fig. S1**).

**Fig. 1:**
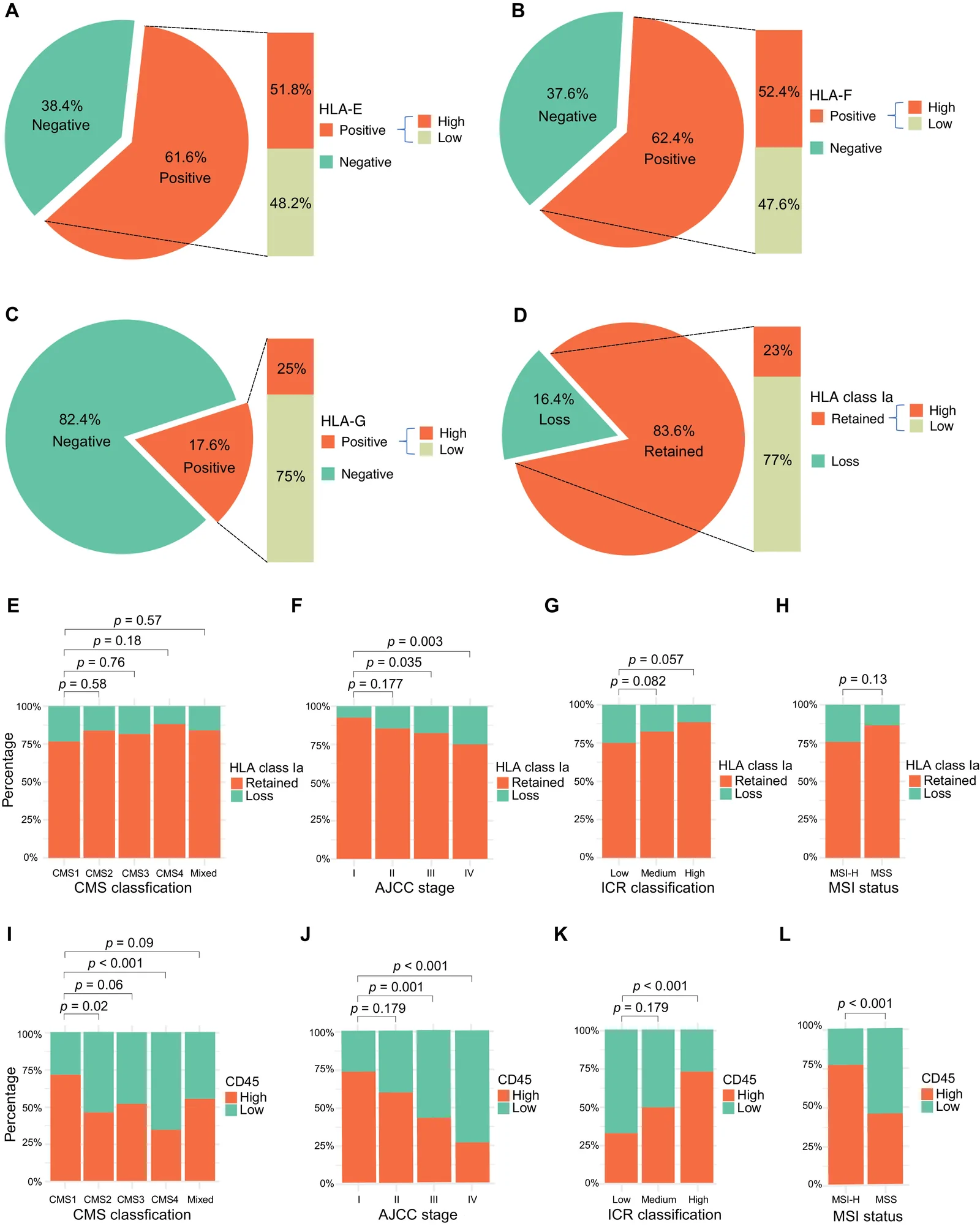
Distribution of HLA class Ia, HLA-E, HLA-F, HLA-G expression and CD45-positive cell density across colon cancer pathological and genomic signatures. (A–C) Pie charts show the proportion of tumors negative vs positive for HLA-E (A), HLA-F (B), and HLA-G (C). The adjacent stacked bars further split the positive cases into high and low intensity categories. (D The pie shows HLA class Ia proficient vs deficient status; the stacked bar depicts the composition of the deficient group (low vs negative). Percentages refer to the proportion of cases in the full cohort; scoring criteria are detailed in Methods. (E–H) showing the distribution of HLA class Ia expression across CMS subtypes (E), AJCC tumor stages (F), ICR scores (G), and MSI status (H). (I–L) showing the distribution of CD45-positive cell density across CMS subtypes (I), AJCC stages (J), ICR scores (K), and MSI status (L). CD45-positive cell density was determined as the number of CD45-positive cells per mm² in annotated tumor epithelium and classified as high and low groups using the median value. P-values indicate statistical significance from pairwise comparisons using Fisher’s exact test; only selected comparisons are shown.

We first characterized the distribution of HLA class Ia and class Ib molecules and the density of intraepithelial CD45-positive cells in the cohort (n = 280) using pre-specified operational definitions (see Methods). HLA-E and HLA-F were Positive in 61.6% and 62.4% of tumors, respectively, with a near-even split between High and Low intensity within the Positive fraction (HLA-E: 51.8% vs. 48.2%; HLA-F: 52.4% vs. 47.6%). HLA-G positivity was less frequent (17.6%), and was predominantly Low (75% of Positive) (Fig. 1A–C). For HLA class Ia, staining in tumor epithelium was quantified with a pathologist-guided, semi-automated image-analysis workflow (**Fig. S2**). Pre-specified coverage cut-offs were applied to the epithelial positive area : Loss if <25% and Retained if ≥25%. Within Retained, coverage was further described as Low (25-<75%) or High (≥75%) (**Fig. S3A-C**). The binary Loss vs Retained variable served as the primary endpoint and used for downstream analyses; the Low/High split is reported for compositional context. Overall, Retained was observed in 83.6% and Loss in 16.4% of tumors; among Retained cases, Low coverage was predominated (77%), while High accounted for 23% (Fig. 1D, side bar). Thus, complete/near-complete Loss of HLA class Ia is less common than Retained with low epithelial coverage in this cohort. The fraction of HLA class Ia Retained varied by CMS, being lowest in CMS1 (i.e., Loss most enriched), whereas CMS2–4 and CMS-Mixed showed higher Retained, with CMS4 displaying the highest Retained proportion (Fig. 1E). Retained decreased monotonically from stage I to stage IV, with the largest Loss fraction at stage IV, consistent with reduced antigen-presentation in advanced disease (Fig. 1F). By immune activity, ICR classification showed a monotonic pattern with higher Retained in ICR-High (Fig. 1G). In line with immune pressure biology, MSI-H tumors showed a shift toward Loss compared to MSS (Fig. 1H). CD45-positive cell density was dichotomized at the cohort median within annotated tumor epithelium (**Fig. S3D-G**). CD45-High tended to be more frequent in CMS1 (Fig. 1I), decreased with increasing AJCC stage (Fig. 1J), rose with ICR (Fig. 1K), and was enriched in MSI-H tumors (Fig. 1L), consistent with an immune-active tumor microenvironment.

### High expression of HLA class Ia and increased CD45-positive cell density associate with improved survival

**Fig. 2:**
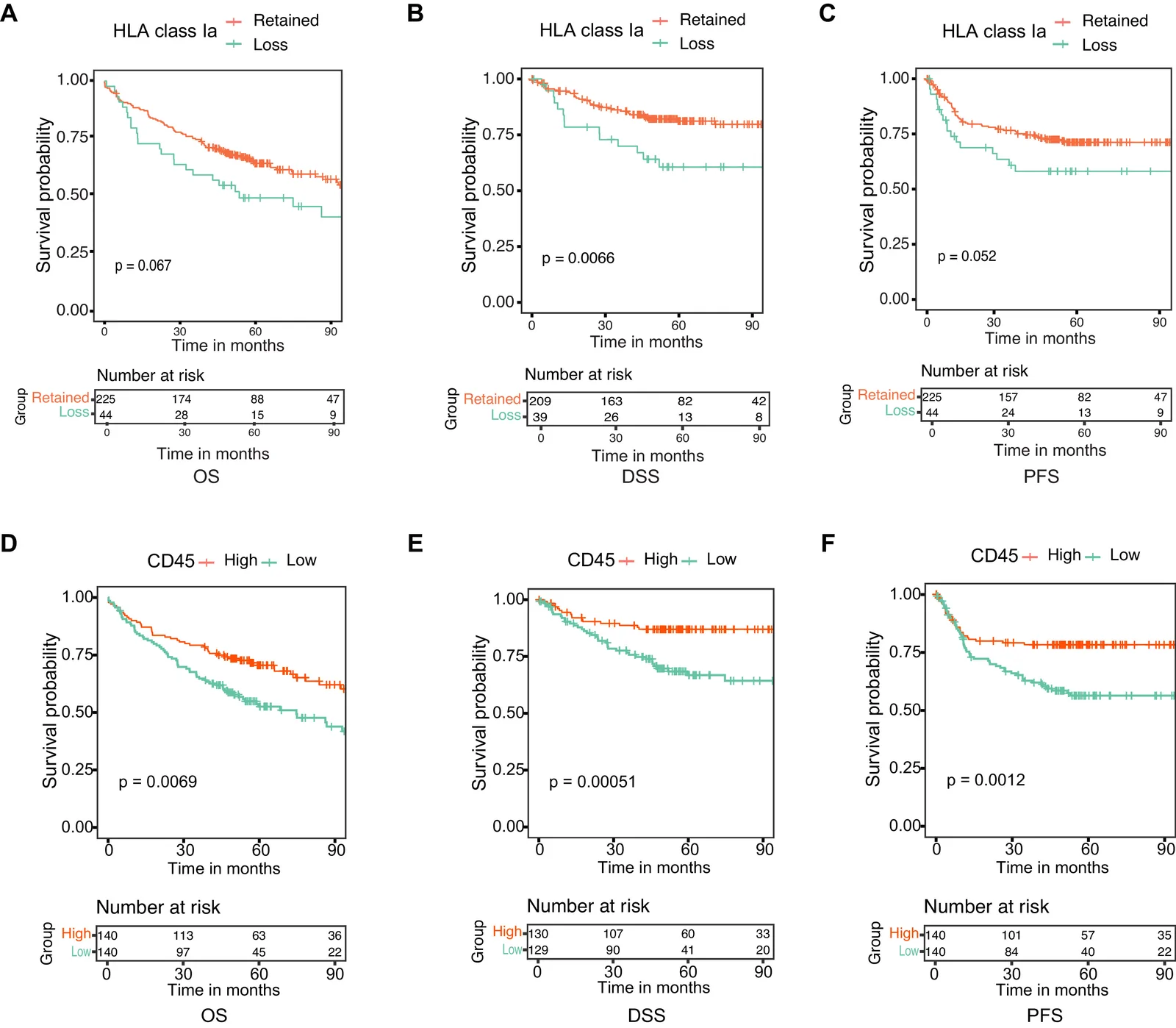
Association of HLA class Ia expression and CD45-positive cell density with patient survival. (A–C) Overall survival (OS), disease-specific survival (DSS), and progression-free survival (PFS) according to HLA class Ia expression. (D–F) OS, DSS, and PFS according to CD45-positive cell density. Group definitions are described in the Methods section. Vertical lines indicate censor points. *P* values are two-sided.

High HLA class Ia expression was significantly associated with disease-specific survival (DSS), and progression-free survival (PFS) (Fig. 2A–C). Similarly, patients with high densities of intraepithelial CD45-positive cells had longer survival at all endpoints (Fig. 2D–F), suggesting enhanced antitumor immunity and improved immune-mediated tumor control.

**Fig. 3:**
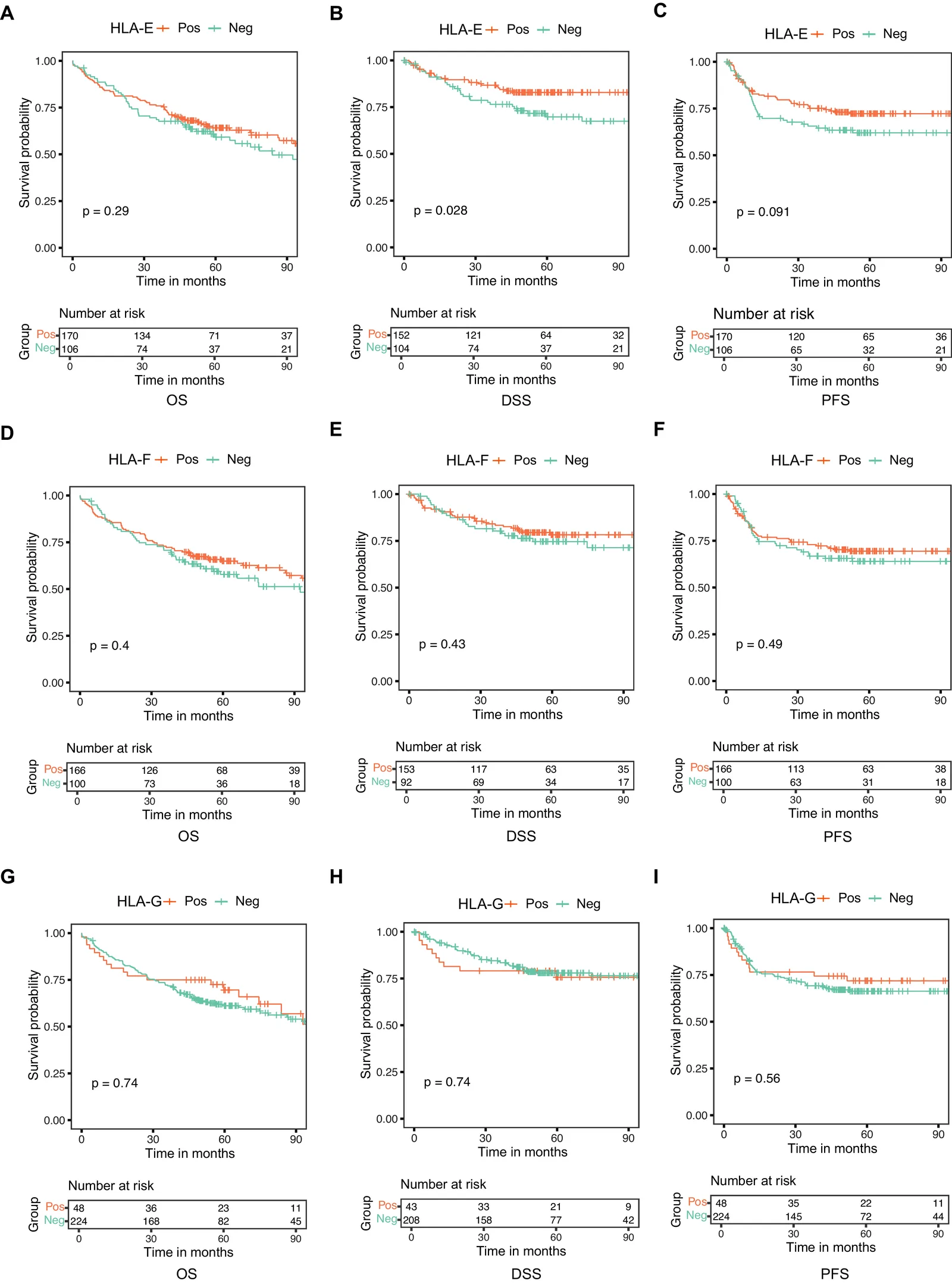
Survival curves stratified by HLA-E, HLA-F, HLA-G tumor expression in colon cancer. (A–C) HLA-E expression and its association with overall survival (OS), disease-specific survival (DSS), and progression-free survival (PFS); (D–F) HLA-F expression and survival outcomes (OS, DSS, PFS); (G–I) HLA-G expression and survival outcomes (OS, DSS, PFS). Vertical lines indicate censor points. *P* values are two-sided.

We also investigated the relationship between HLA class Ib molecule expression and patient survival (Fig. 3). High HLA-E expression was associated with improved DSS and PFS (Fig. 3B and C). Moreover, an association was observed between HLA class Ia and HLA-E expression levels (**Fig. S5**, p < 0.05), supporting the role of HLA class Ia in stabilizing HLA-E surface presentation through signal peptides provision [37]. Expression of HLA-F and HLA-G showed no significant association with survival outcomes (Fig. 3D–I).

### HLA class Ib and immune activity in colon cancer

**Fig. 4:**
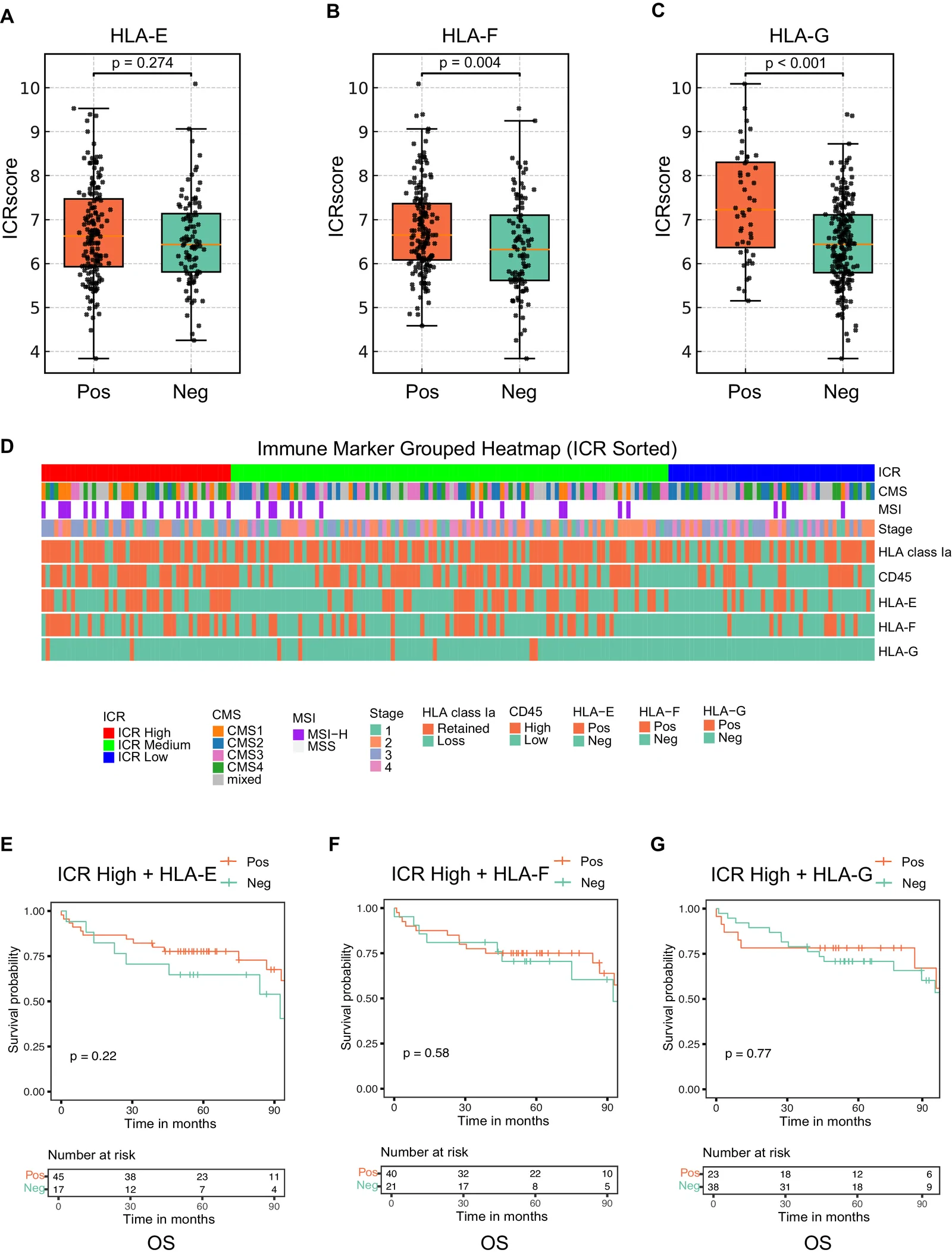
ICR score distributions stratified by expression levels of HLA class Ib molecules. (A) HLA-E, (B) HLA-F, and (C) HLA-G expression grouped as Postiive or Negative. ICR scores are plotted as continuous variables across these categories. Boxes represent interquartile ranges; whiskers indicate 1.5× interquartile range (IQR); individual points represent samples. D. Overview of the distribution of HLA class Ia, class Ib expression and CD45-positive cell density in relation to genomic signatures. Kaplan–Meier overall survival analysis stratified by HLA-E (E), HLA-F (F) or HLA-G (G) expression levels in tumors with high ICR scores. Expression was grouped as Positive or Negative. Vertical lines indicate censor points. *P* values are two-sided.

We next evaluated the relationship between HLA class Ib molecule expression and immune activity by comparing ICR scores across tumors stratified by HLA-E, HLA-F, and HLA-G expression. When tumors were grouped by HLA class Ib status (Positive vs Negative), HLA-F and HLA-G showed higher ICR scores in Positive cases, whereas HLA-E did not differ significantly (Fig. 4A–C). Further analysis revealed that HLA-E and HLA-F molecules were more frequently expressed in tumors with high ICR scores, paralleling the expression patterns of HLA class Ia and CD45-positive cell density (Fig. 4D). These findings demonstrate that HLA class Ib expression is associated with a more immune-active tumor microenvironment.

To assess whether the expression of HLA class Ib molecules affects the survival rate of immunologically active tumors, we conducted a stratified survival analysis in the ICR-high subgroup. Kaplan–Meier analysis showed that in ICR-high tumors, expression of HLA-E, HLA-F or HLA-G was not significantly associated with overall survival (Fig. 4E–G). Disease-specific survival (DSS) and progression-free survival (PFS) analyses yielded similar results, with no significant differences observed between HLA-E, HLA-F or HLA-G expression groups in the ICR-high cohort (**Fig. S6**). These findings suggest that while HLA class Ib molecules are associated with immune activity, they do not independently influence clinical outcomes in tumors already enriched with immune activity.

### Tumor immune phenotype is associated with survival

**Fig. 5:**
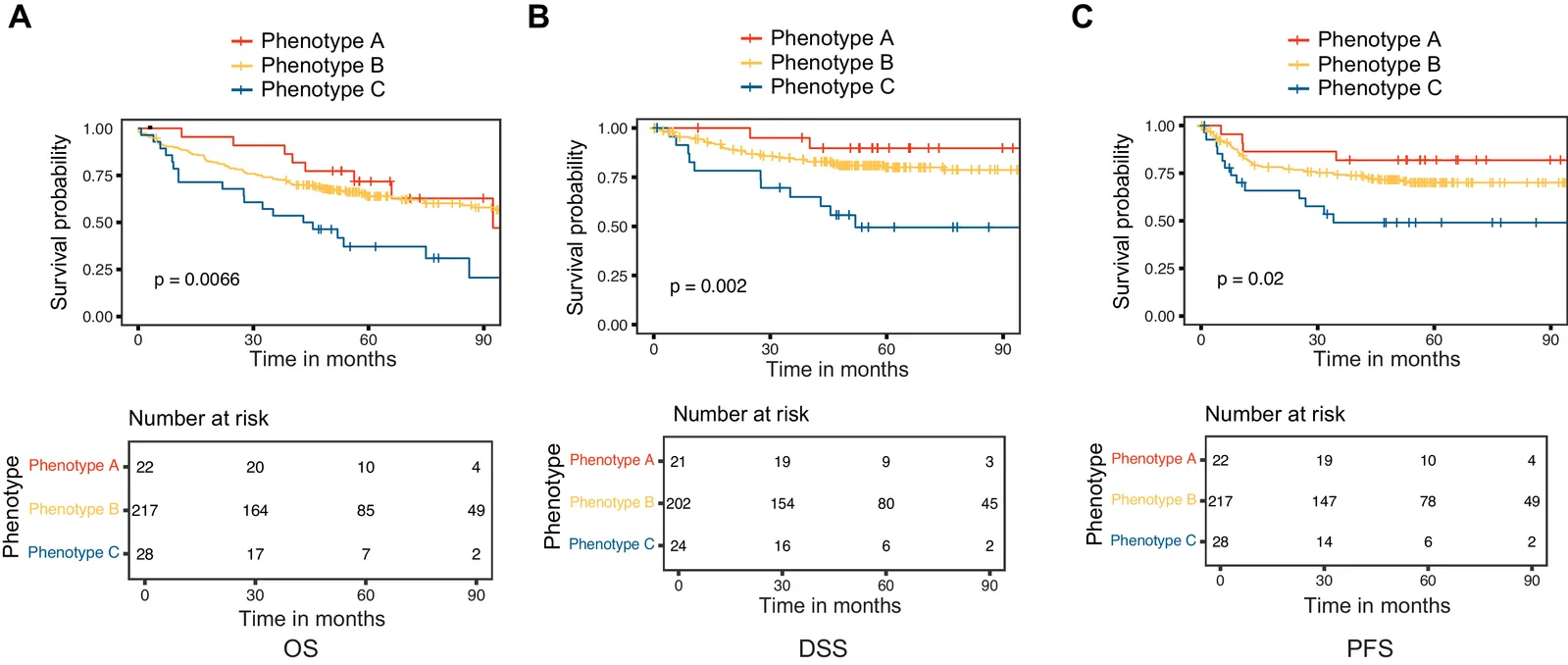
Survival curves stratified by immune phenotypes in colon cancer. (A–C) Overall survival (OS), disease-specific survival (DSS), and progression-free survival (PFS), stratified by three composite immune phenotypes (see Methods). OS: n = 267; DSS: n = 247; PFS: n = 267. Vertical lines indicate censor points. *P* values are two-sided.

Although both HLA class Ia expression and CD45-positive immune cell density were individually associated with improved survival, the interplay between HLA class Ia and Ib molecules and immune infiltration may shape distinct tumor immune phenotypes. To better capture this complexity, we analyzed survival outcomes based on combined expression profiles of HLA class Ia, HLA class Ib molecules (HLA-E, -F, and -G) and CD45-positive cell density, and identified three immune phenotypes with distinct outcome implications (Fig. 5**, Fig. S7**). The first phenotype (Phenotype A) was characterized by high expression of HLA class Ia and high CD45-positive cell density and absence of expression of HLA-E, HLA-F and HLA-G (n = 22). The second phenotype (Phenotype B) includes tumors that either (i) show high expression of HLA class Ia or CD45-positive cell density alone (but not both), or (ii) show both high expression of HLA class Ia and CD45-positive cell density together with high expression of HLA class Ib (n = 217). The third phenotype (Phenotype C) is characterized by low HLA class Ia expression and low CD45-positive cell density, regardless of HLA class Ib status (n = 28).

These combined immune features were associated with significantly different survival outcomes, suggesting that the combination of immune infiltration and HLA class Ia and Ib tumor expression provides a more comprehensive indicator of patient outcome. We also performed a Cox proportional hazards multivariate analysis to assess factors associated with recurrence over time (Table 1). The multivariate model included the following variables: adjuvant therapy, AJCC stage, ICR classification, HLA class Ia expression, CD45-positive cell density, and immune phenotype (a combined variable of immune markers). The results of the analysis showed that AJCC stage and immune phenotype were independent and important predictors of OS. Interestingly, although the importance of HLA class Ia expression and CD45-positive cell density is well known, they did not retain independent significance in the multivariate model analysis, possibly because their combined effect was better reflected in the immune phenotype classification. This finding further supports the concept that the integration of multiple immune markers into a composite immune phenotype more robustly classifies risk than the individual assessment of single markers.

**Table 1:** Univariate and multivariate outcome analyses for overall survival (OS).

Univariate				Multivariate		
	HR	95% CI	P value	HR	95% CI	P value
**Sex**						
Female	1	0.808-1.646	0.431			
Male	1.153					
**Anatomical location**						
Left sided	1		0.62			
Right side	1.094	0.767-1.559				
**Adjuvant treatment**				1		
No	1		0.599	1.247	0.646-2.410	
Yes	1.106	0.759-1.613		1.367	0.692-2.701	
**AJCC stage**				5.226	2.637-10.360	
I	1		**<0.001**			**<0.001**
II	1.228	0.640-2.358	0.536			0.649
III	1.451	0.749-2.810	0.27			0.421
IV	5.505	0.883-10.51	**<0.001**			**<0.001**
**MSI status**						
MSS	1		0.214			
MSI-High	0.814	0.523-1.129				
**ICR classification**						
Low	1		**0.046**	1		0.223
Medium	0.627	0.418-0.942	**0.025**	0.955	0.550-1.657	0.869
High	0.59	0.355-0.97	**0.041**	0.715	0.471-1.087	0.116
**CMS classification**						
CMS1	1		0.09			
CMS2	0.67	0.352-1.27	0.223			
CMS3	0.793	0.414-1.521	0.486			
CMS4	1.39	0.759-2.544	0.286			
Mixed	1.046	0.574-1.90	0.883			
**HLA-Ia expression**						
Low	1		**0.027**	1		
High	0.67	0.469-0.956		0.801	0.564-1.208	0.252
**CD45 density**						
Low	1		**0.007**	1		
High	0.614	0.429-0.877		0.889	0.602-1.372	0.567
**Immune phenotypes**						
Phenotype A	1		**<0.001**	1		**0.045**
Phenotype B	2.6	2.9-11.3		2.3	1.7-9.6	
Phenotype C	5.8	4.1-16.7		4.4	2.1-12.4	

Hazard ratios (HR), 95% confidence intervals (CI), and P values are shown for each variable. AJCC: American Joint Committee on Cancer; MSI: Microsatellite instability; ICR: Immunologic Constant of Rejection classification; CMS: Consensus Molecular Subtypes. Bold values indicate statistically significant results (*P* < 0.05).

## Discussion

In this study, we conducted an integrated analysis of HLA class Ia, CD45, and HLA class Ib molecules (HLA-E, HLA-F, HLA-G) in colon cancer to assess the association of these molecules with clinical-pathological variables, MSI status, CMS, ICR classification, and patient outcomes. The findings highlight the importance of antigen presentation capacity and immune cell infiltration in shaping the tumor immune microenvironment. Although classification frameworks, such as MSI [38] and ICR [1], have significantly advanced our understanding of tumor immunobiology, integrating multiple immune markers covering also HLA class Ia and class Ib pathways, may provide a complementary view of immune microenvironment diversity, particularly regarding antigen presentation and immune cell engagement. The immune phenotypes identified in this study were associated with differences in patient outcomes, suggesting their importance in future studies aimed at optimizing immune classification and stratification strategies.

Across studies, findings for HLA immunohistochemistry are discrepant. Differences in HLA IHC across studies can plausibly account for discrepant results, including antibody clones/epitopes (e.g., EMR8-5 vs HCA2/HC10/W6/32 for class Ia [33,39]; MEM-E/02 vs MEM-E/07, 3D11 vs EPR6803, 4H84 vs MEM-G/1 for Ib [20,40,41]), retrieval/detection chemistry and chromogen order in double stains [42], the compartment/readout scored (membranous coverage vs H-score or epithelium vs mixed [43,44]), and cut-off definitions [45,46]. In this study we mitigated variability by using a standardized protocol with fixed chromogen order, quantifying tumor epithelial coverage for HLA class Ia, restricting Ib reads to tumor epithelial patterns, and applying blinded dual review (κ>0.8) with complete-case analyses and explicit analysis subsets. Our results showed that retained HLA class Ia expression was largely uniform across AJCC stages I–III, with only a modest shift toward Loss in stage IV. This pattern may reflect the selective outgrowth of tumor clones with reduced antigen-presenting capacity during advanced disease progression. This observation is also consistent with previous studies, which have confirmed that HLA class Ia loss is a common immune escape strategy that enables tumors to evade CD8+ T cell-mediated cytotoxicity [[47], [48], [49]]. In addition, a significant positive correlation between HLA class Ia expression and ICR was observed, which supports the view that HLA class Ia plays a key role in maintaining an active anti-tumor immune response, as tumors with higher ICR scores exhibited stronger retained HLA class Ia expression [50]. Interestingly, retained HLA class Ia expression in MSI-H tumors was lower than in MSS tumors. MSI-H tumors are often associated with strong immune activation due to increased neoantigen load, and the loss of HLA class Ia may reflect an adaptive immune escape mechanism. Consistent with previous reports [26], this loss of expression may be due to selective pressure in the inflammatory tumor microenvironment, allowing tumor cells to evade CD8⁺ T cell-mediated cytotoxicity through impaired antigen presentation. Notably, our readout quantified pan–HLA class Ia heavy chain with EMR8-5 and did not directly assess β2-microglobulin (B2M). Given that truncating B2M alterations are frequent in MSI-H colon cancer and can abolish functional surface HLA-I despite residual heavy-chain signal detected by IHC [18], the modest decrease in HLA-Ia membranous staining we observe in MSI-H tumors likely underestimates the frequency of complete functional loss of HLA-I, especially in B2M-deficient tumors.

Similar to HLA class Ia, the density of CD45-positive cells decreases with increasing AJCC stage, which may reflect reduced immune infiltration in advanced tumors or that tumors with lower immune infiltration are more prone to progression. The strong correlation between CD45-positive cell density and ICR score indicates that tumors with an active immune environment also exhibit stronger immune cell infiltration, which is consistent with the role of ICR as a marker of effector immune response. The density of CD45-positive cells in MSI-H tumors was significantly higher than in MSS tumors, which is consistent with previous reports describing the presence of a large number of immune infiltrates in MSI-H tumors [51,52].

The association observed between retained HLA class Ia expression, increased infiltrating CD45-positive cell density, and favorable survival outcomes further emphasizes the importance of antigen presentation and active immune infiltration in effective tumor control. These findings are consistent with previous studies and suggest that immune activation is a key determinant of long-term clinical benefit in colon cancer [24,53]. In contrast, the lack of a clear association between HLA-F or HLA-G expression and survival suggests that these HLA class Ib molecules may play a more complex or context-dependent role, possibly regulating the immune response under specific microenvironmental conditions. We also note that interpretation of HLA-G should be cautious because the 4H84 monoclonal antibody has been reported to yield non-HLA-G-specific staining under certain pre-analytical or protocol conditions [20,54]. In our study, we sought to mitigate this by standardized retrieval and detection, restricting scoring to tumor epithelial patterns with dual-review adjudication and external positive/negative controls, but residual non-HLA_G-specificity cannot be fully excluded and may have diluted true associations.

Interestingly, HLA-E expression was associated with improved DSS and PFS, a finding that contrasts with its traditional immunosuppressive role via NKG2A/CD94-mediated suppression of NK and CD8+ T cells [[55], [56], [57], [58]]. This unexpected result suggests that HLA-E may also promote anti-tumor immunity under certain conditions, possibly by presenting antigenic peptides to unconventional T cell subsets such as NKG2C-expressing CD8+ T cells [59,60]. It is important to note that the stability and peptide loading of HLA-E are significantly influenced by signal peptides from HLA class Ia molecules, with variations in these sequences among certain HLA-I alleles affecting HLA-E's peptide affinity [41,61,62]. This allele-dependent peptide supply to HLA-E suggests that the surface levels and function of HLA-E are partially dependent on the existing HLA class Ia landscape, rather than functioning independently. Reduced expression of HLA class Ia in tumors could limit the availability of leader peptides and decrease HLA-E stabilization [62], but inflammatory factors such as IFN-γ or other peptide sources can preserve HLA-E and direct signaling towards inhibition via NKG2A [[62], [63], [64]]. These interconnections provide a reasonable explanation for what we observed that elevated levels of HLA-E were linked to enhanced DSS/PFS, implying that HLA-E could identify an immune-active setting in certain cases, where unconventional CD8+T cells aid in anti-tumor responses.

The integration of multiple immune parameters revealed distinct immune phenotypes in colon cancer that are independently associated with clinical outcomes. This finding is consistent with recent studies, emphasizing the prognostic value of combined immune characteristics rather than single markers [17,19]. For example, previous studies have shown that tumors with abundant T cell infiltration and retained HLA class Ia expression tend to exhibit more active immune response characteristics, such as increased expression of cytotoxic genes or inflammatory markers [[65], [66], [67]]. In the present cohort, tumors with retained HLA class Ia expression and elevated CD45-positive cell density exhibited an immune-active phenotype, while tumors lacking both features were consistent with an immune-exhausted state, which has previously been associated with the stromal barrier, inadequate T cell priming, and resistance to checkpoint blockade therapy [[68], [69], [70], [71]]. Notably, the intermediate group (only one of the two key components, retained HLA class Ia expression or elevated CD45-positive cell density) suggests heterogeneous immune states, potentially representing tumors with partial immune activation or adaptive resistance. These phenotypes provide a practical framework for optimizing risk stratification and may help explicate the underlying immune response beyond microsatellite instability status.

Despite the strengths of this study, its limitations should be considered. First, a prospective validation in an independent patient cohort is necessary to confirm the reliability of the immunophenotypic classification in the future. Second, although our findings demonstrate an association between immune markers and patient outcomes, the underlying mechanisms remain to be elucidated. Functional studies are needed to explore how HLA class Ia loss, HLA-E-mediated immune regulation, and immune rejection contribute to tumor progression in colon cancer. In addition, the integration of other immune parameters, such as immune checkpoint expression, tumor mutational load, and spatial immune analysis, may further optimize immunophenotypical classification and enhance its clinical relevance. Understanding these interactions may ultimately help develop more effective immunotherapy strategies targeting specific immune phenotypes.

## Conclusion

In summary, our study highlights the relevance of HLA class Ia/Ib and immune cell infiltration in affecting the outcome of colon cancer. By combining multiple immune markers, we identified unique immune phenotypes that reflect the diversity of tumor-immune interactions and correlate with differences in patient survival. These findings contribute to a better understanding of immune escape and control mechanisms in colon cancer and lay the foundation for future prospective studies aimed at validating these phenotypes. In particular, their potential relevance in immunotherapy warrants further investigation.

## Declarations

### Ethics approval and consent to participate

The collected patient information was de-identified and the tissue samples were handled in accordance with the guidelines outlined in the Code of Conduct for Proper Secondary Use of Human Tissue of the Dutch Federation of Biomedical Scientific Societies, ensuring anonymity. The study was conducted in accordance with the principles described in the Helsinki Declaration and was approved by the IRB at LUMC and Sidra Medicine (IRB: 1768087-1, 1602002725 and B19.079). This study is reported in accordance with the STROBE guidelines (cohort).

### Consent for publication

Not applicable. This study does not contain any individual person’s identifiable data, including images or clinical details.

## Funding

Authors HZ was supported by a CSC (China Scholarship Council) grant.

## CRediT authorship contribution statement

**Hehuan Zhu:** Data curation, Formal analysis, Investigation, Software, Validation, Visualization, Writing – original draft, Writing – review & editing. **Roos van Nieuwkerk:** Data curation, Formal analysis, Investigation, Software, Writing – original draft. **Savannah van der Kroft:** Formal analysis, Methodology, Validation, Writing – original draft. **Ronald L.P. van Vlierberghe:** Investigation, Methodology, Writing – original draft. **Marieke E. IJsselsteijn:** Investigation, Methodology, Validation, Writing – original draft, Writing – review & editing. **Weimin Zuo:** Investigation, Visualization, Writing – review & editing. **Thomas Vauvert F. Hviid:** Methodology, Resources, Supervision, Writing – review & editing. **Rob A.E.M. Tollenaar:** Funding acquisition, Project administration, Resources, Supervision, Writing – review & editing. **Alexander L. Vahrmeijer:** Funding acquisition, Project administration, Supervision, Writing – review & editing. **Davide Bedognetti:** Methodology, Project administration, Supervision, Writing – review & editing. **Wouter R.L. Hendrickx:** Investigation, Project administration, Resources, Supervision, Writing – review & editing. **Jessica Roelands:** Conceptualization, Data curation, Investigation, Project administration, Software, Supervision, Validation, Writing – original draft, Writing – review & editing. **Peter J.K. Kuppen:** Conceptualization, Formal analysis, Funding acquisition, Project administration, Supervision, Visualization, Writing – original draft, Writing – review & editing.

## Declaration of competing interest

The authors declare that they have no known competing financial interests or personal relationships that could have appeared to influence the work reported in this paper.

## Data Availability

Data was available in the Supplementary Source Data File corresponding to the original publication [1] (Gene expression matrix in Supplementary Source Data 3, CMS classification in Supplementary Source Data 4, ICR clusters in Supplementary Source Data 19, and MSI in Supplementary Source Data 11).

## Appendix Supplementary materials

Image, application 1

Image, application 2

Image, application 3

Image, application 4

Image, application 5

Image, application 6

Image, application 7

Image, application 8

Image, application 9
