# Supplementary material for: HLA class I expression and tumor immune infiltration together shape colon cancer immune contexture
Source: Neoplasia. 2026 Sep 2;81:101357. doi: 10.1016/j.neo.2026.101357 (PMC13570313; doi:10.1016/j.neo.2026.101357)
Supplement: Supplementary file 1 [file mmc1.pdf]

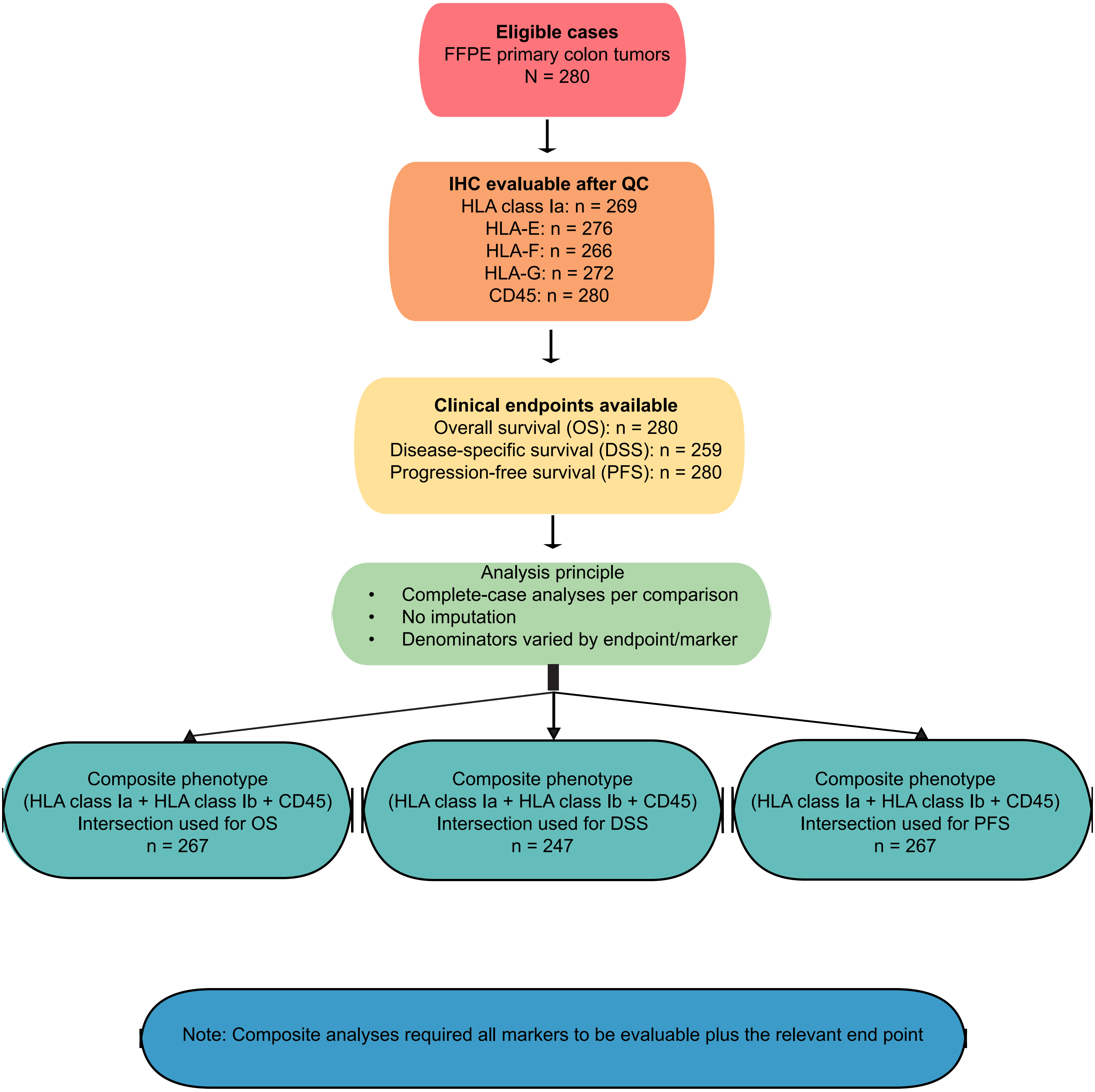

Eligible cases
FFPE primary colon tumors
N = 280
IHC evaluable after QC
HLA class Ia: n = 269
HLA-E: n = 276
HLA-F: n = 266
HLA-G: n = 272
CD45: n = 280
Clinical endpoints available
Overall survival (OS): n = 280
Disease-specific survival (DSS): n = 259
Progression-free survival (PFS): n = 280
Analysis principle
• Complete-case analyses per comparison
• No imputation
• Denominators varied by endpoint/marker
Composite phenotype
(HLA class Ia + HLA class Ib + CD45)
Intersection used for OS
n = 267
Composite phenotype
(HLA class Ia + HLA class Ib + CD45)
Intersection used for DSS
n = 247
Composite phenotype
(HLA class Ia + HLA class Ib + CD45)
Intersection used for PFS
n = 267
Note: Composite analyses required all markers to be evaluable plus the relevant end point
