# Supplementary material for: HLA class I expression and tumor immune infiltration together shape colon cancer immune contexture
Source: Neoplasia. 2026 Sep 2;81:101357. doi: 10.1016/j.neo.2026.101357 (PMC13570313; doi:10.1016/j.neo.2026.101357)
Supplement: Supplementary file 2 [file mmc2.pdf]

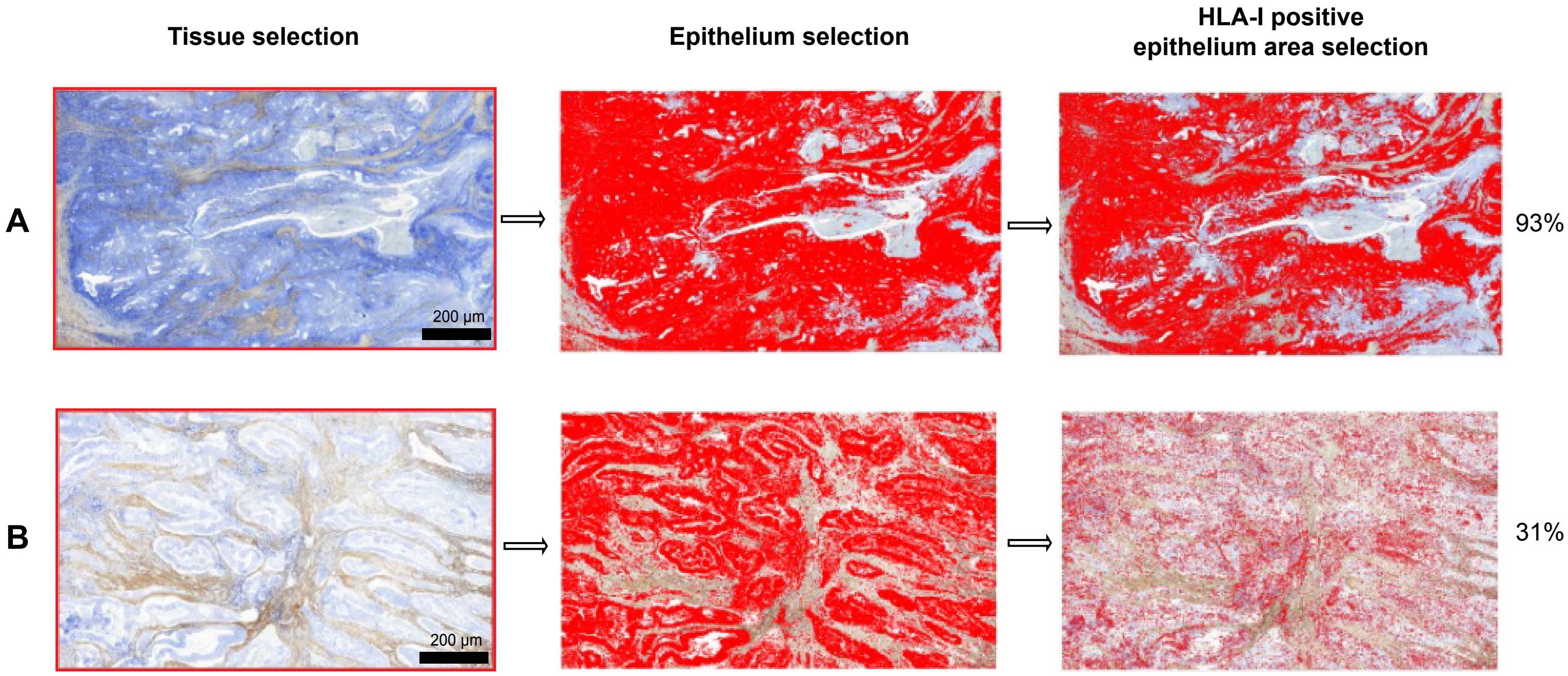
Tissue selection
Epithelium selection
HLA-I positive
epithelium area selection
A
200 µm
93%
B
200 µm
31%
