# Supplementary material for: HLA class I expression and tumor immune infiltration together shape colon cancer immune contexture
Source: Neoplasia. 2026 Sep 2;81:101357. doi: 10.1016/j.neo.2026.101357 (PMC13570313; doi:10.1016/j.neo.2026.101357)
Supplement: Supplementary file 3 [file mmc3.pdf]

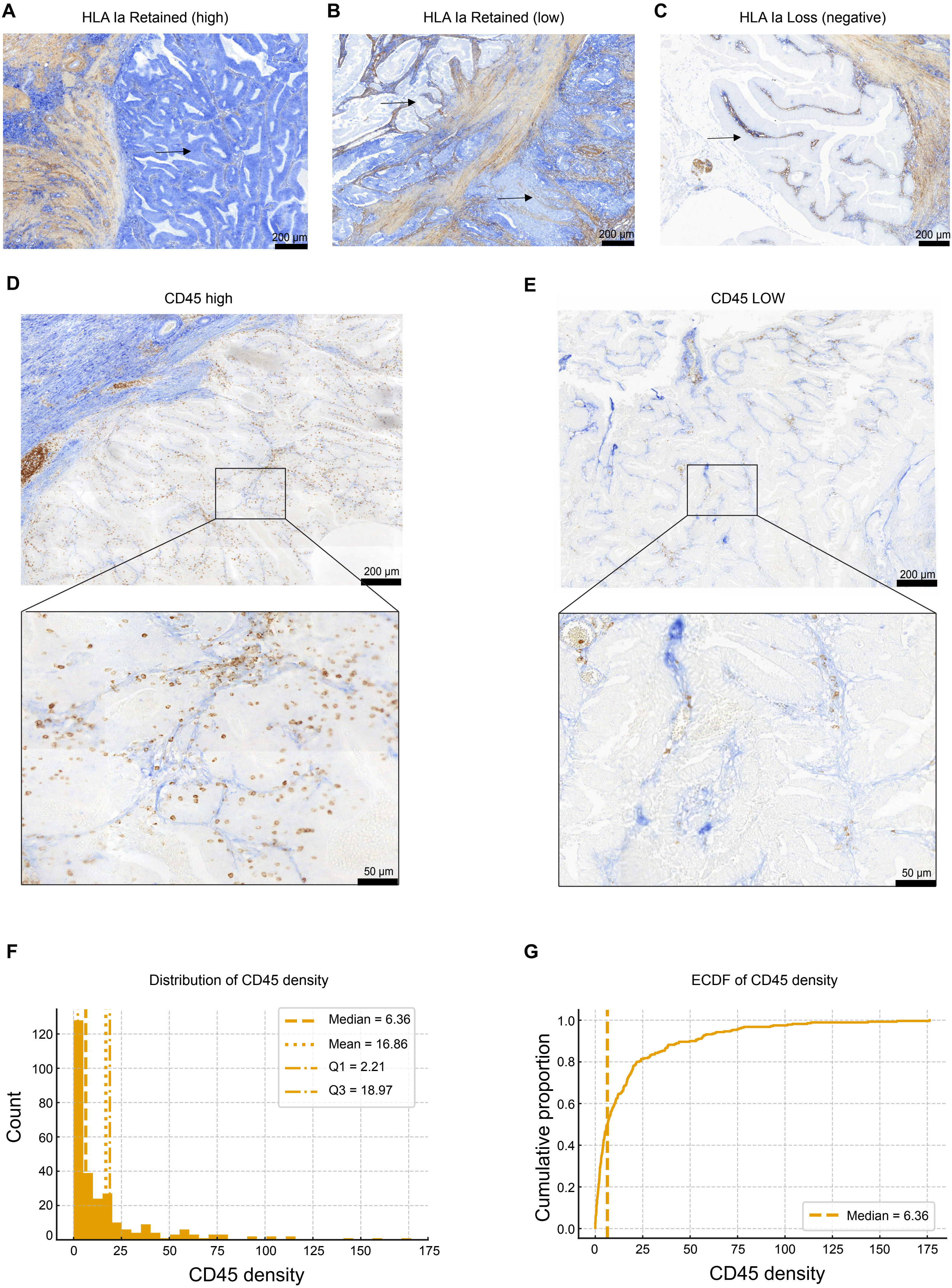
A
HLA Ia Retained (high)
200 µm
B
HLA Ia Retained (low)
200 µm
C
HLA Ia Loss (negative)
200 µm
D
CD45 high
200 µm
50 µm
E
CD45 LOW
200 µm
50 µm
F
Distribution of CD45 density
Median = 6.36
Mean = 16.86
Q1 = 2.21
Q3 = 18.97
Count
CD45 density
G
ECDF of CD45 density
Cumulative proportion
Median = 6.36
CD45 density
