# Supplementary material for: HLA class I expression and tumor immune infiltration together shape colon cancer immune contexture
Source: Neoplasia. 2026 Sep 2;81:101357. doi: 10.1016/j.neo.2026.101357 (PMC13570313; doi:10.1016/j.neo.2026.101357)
Supplement: Supplementary file 4 [file mmc4.pdf]

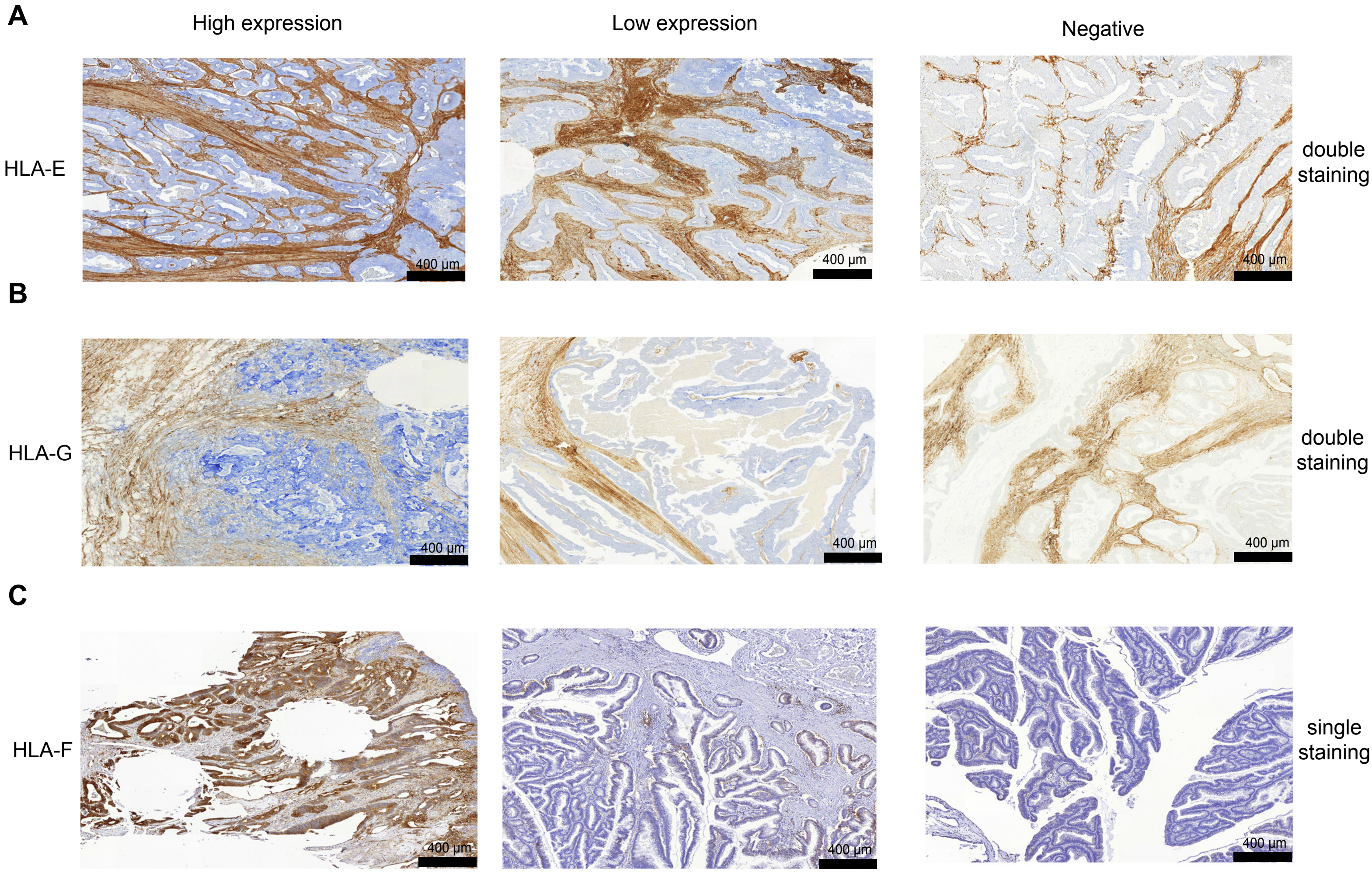
A
High expression
Low expression
Negative
HLA-E
double staining
B
HLA-G
double staining
C
HLA-F
single staining
400 µm
