# Supplementary material for: HLA class I expression and tumor immune infiltration together shape colon cancer immune contexture
Source: Neoplasia. 2026 Sep 2;81:101357. doi: 10.1016/j.neo.2026.101357 (PMC13570313; doi:10.1016/j.neo.2026.101357)
Supplement: Supplementary file 5 [file mmc5.pdf]

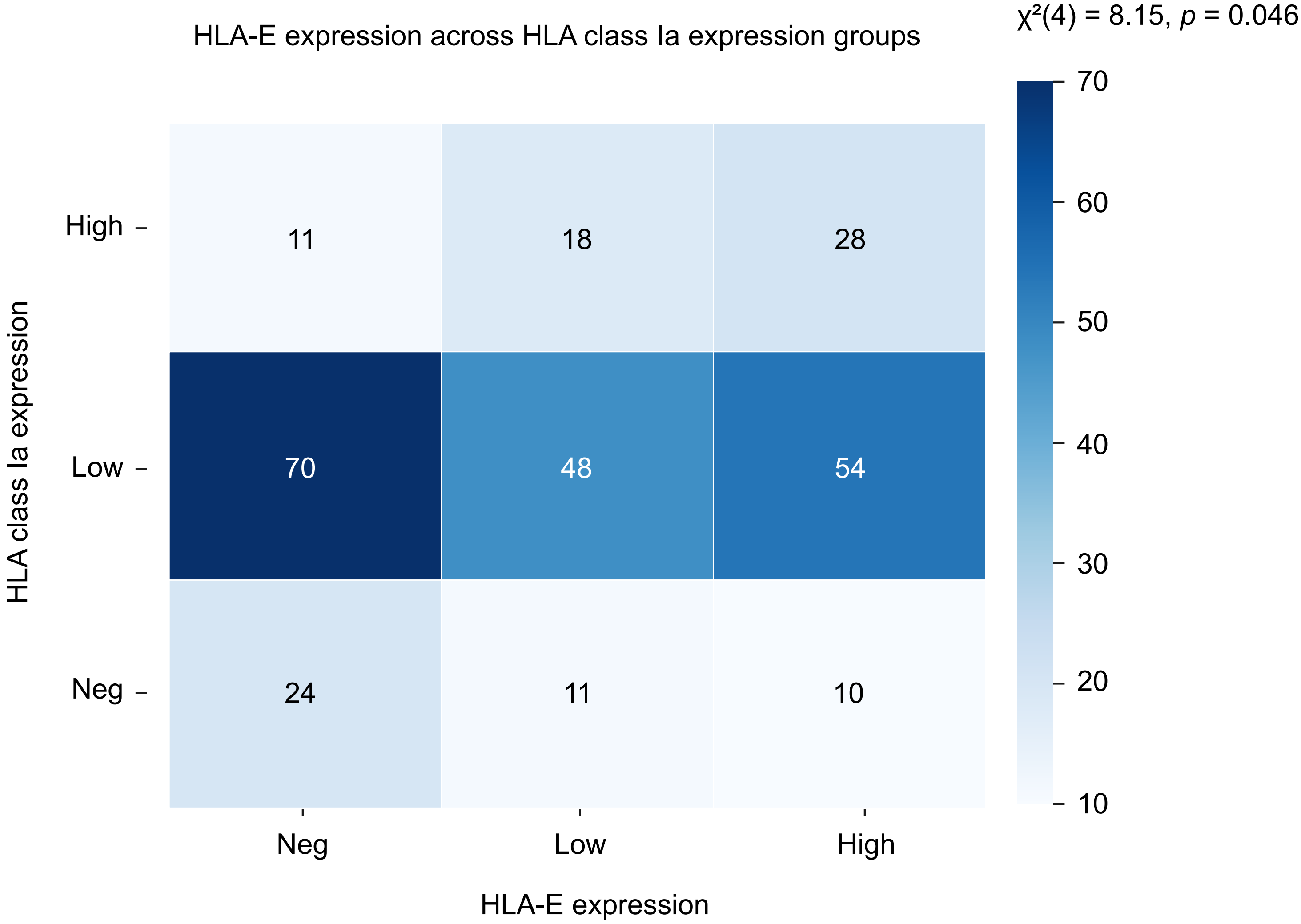
HLA-E expression across HLA class Ia expression groups
χ²(4) = 8.15, p = 0.046
HLA class Ia expression
High
Low
Neg
11
18
28
70
48
54
24
11
10
Neg
Low
High
HLA-E expression
70
60
50
40
30
20
10
