# Supplementary material for: HLA class I expression and tumor immune infiltration together shape colon cancer immune contexture
Source: Neoplasia. 2026 Sep 2;81:101357. doi: 10.1016/j.neo.2026.101357 (PMC13570313; doi:10.1016/j.neo.2026.101357)
Supplement: Supplementary file 6 [file mmc6.pdf]

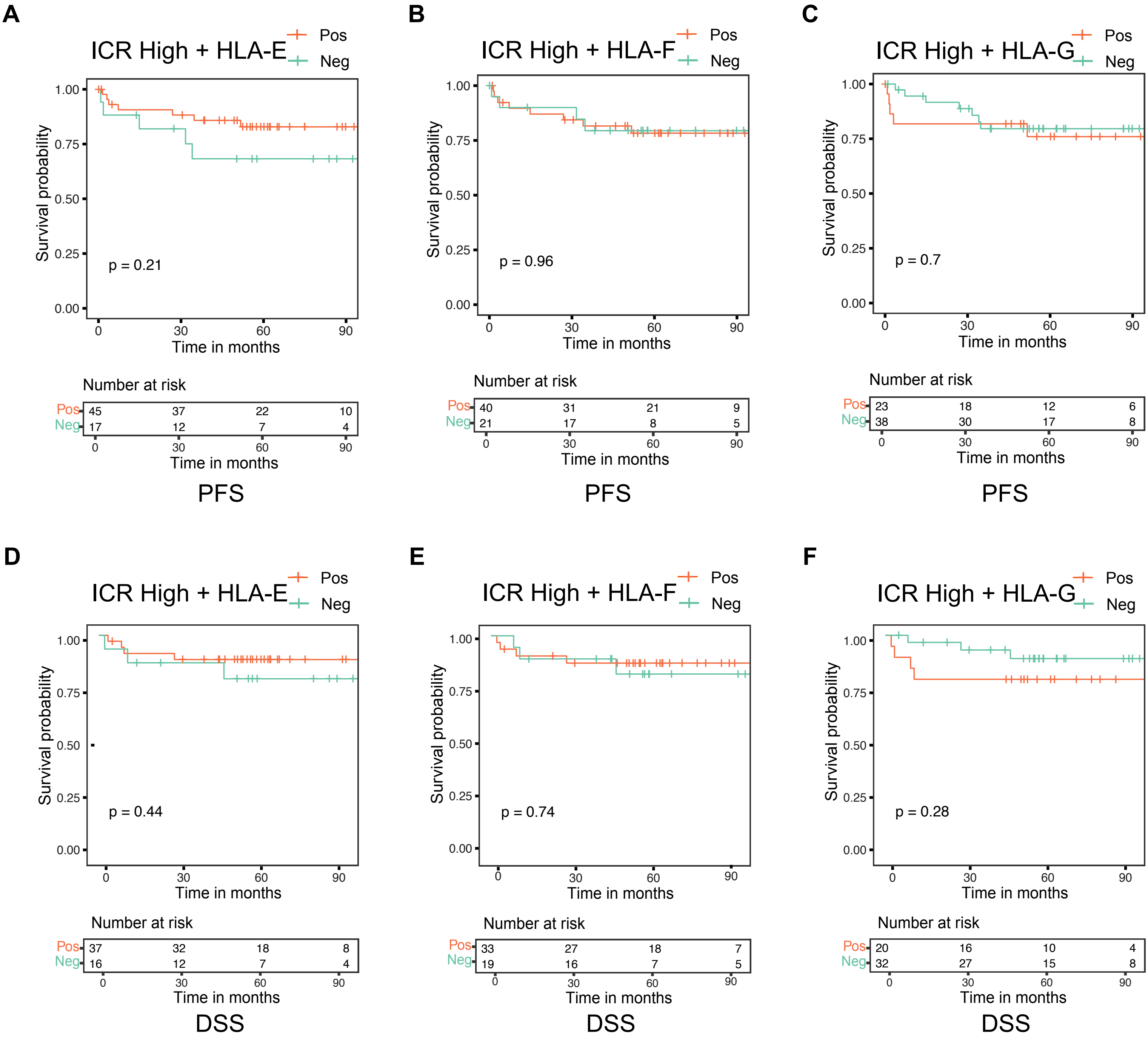

A
ICR High + HLA-E
Pos
Neg
Survival probability
1.00
0.75
0.50
0.25
0.00
p = 0.21
0
30
60
90
Time in months
Number at risk
Pos 45 37 22 10
Neg 17 12 7 4
0
30
60
90
Time in months
PFS
B
ICR High + HLA-F
Pos
Neg
Survival probability
1.00
0.75
0.50
0.25
0.00
p = 0.96
0
30
60
90
Time in months
Number at risk
Pos 40 31 21 9
Neg 21 17 8 5
0
30
60
90
Time in months
PFS
C
ICR High + HLA-G
Pos
Neg
Survival probability
1.00
0.75
0.50
0.25
0.00
p = 0.7
0
30
60
90
Time in months
Number at risk
Pos 23 18 12 6
Neg 38 30 17 8
0
30
60
90
Time in months
PFS
D
ICR High + HLA-E
Pos
Neg
Survival probability
1.00
0.75
0.50
0.25
0.00
p = 0.44
0
30
60
90
Time in months
Number at risk
Pos 37 32 18 8
Neg 16 12 7 4
0
30
60
90
Time in months
DSS
E
ICR High + HLA-F
Pos
Neg
Survival probability
1.00
0.75
0.50
0.25
0.00
p = 0.74
0
30
60
90
Time in months
Number at risk
Pos 33 27 18 7
Neg 19 16 7 5
0
30
60
90
Time in months
DSS
F
ICR High + HLA-G
Pos
Neg
Survival probability
1.00
0.75
0.50
0.25
0.00
p = 0.28
0
30
60
90
Time in months
Number at risk
Pos 20 16 10 4
Neg 32 27 15 8
0
30
60
90
Time in months
DSS
