# Supplementary material for: HLA class I expression and tumor immune infiltration together shape colon cancer immune contexture
Source: Neoplasia. 2026 Sep 2;81:101357. doi: 10.1016/j.neo.2026.101357 (PMC13570313; doi:10.1016/j.neo.2026.101357)
Supplement: Supplementary file 7 [file mmc7.pdf]

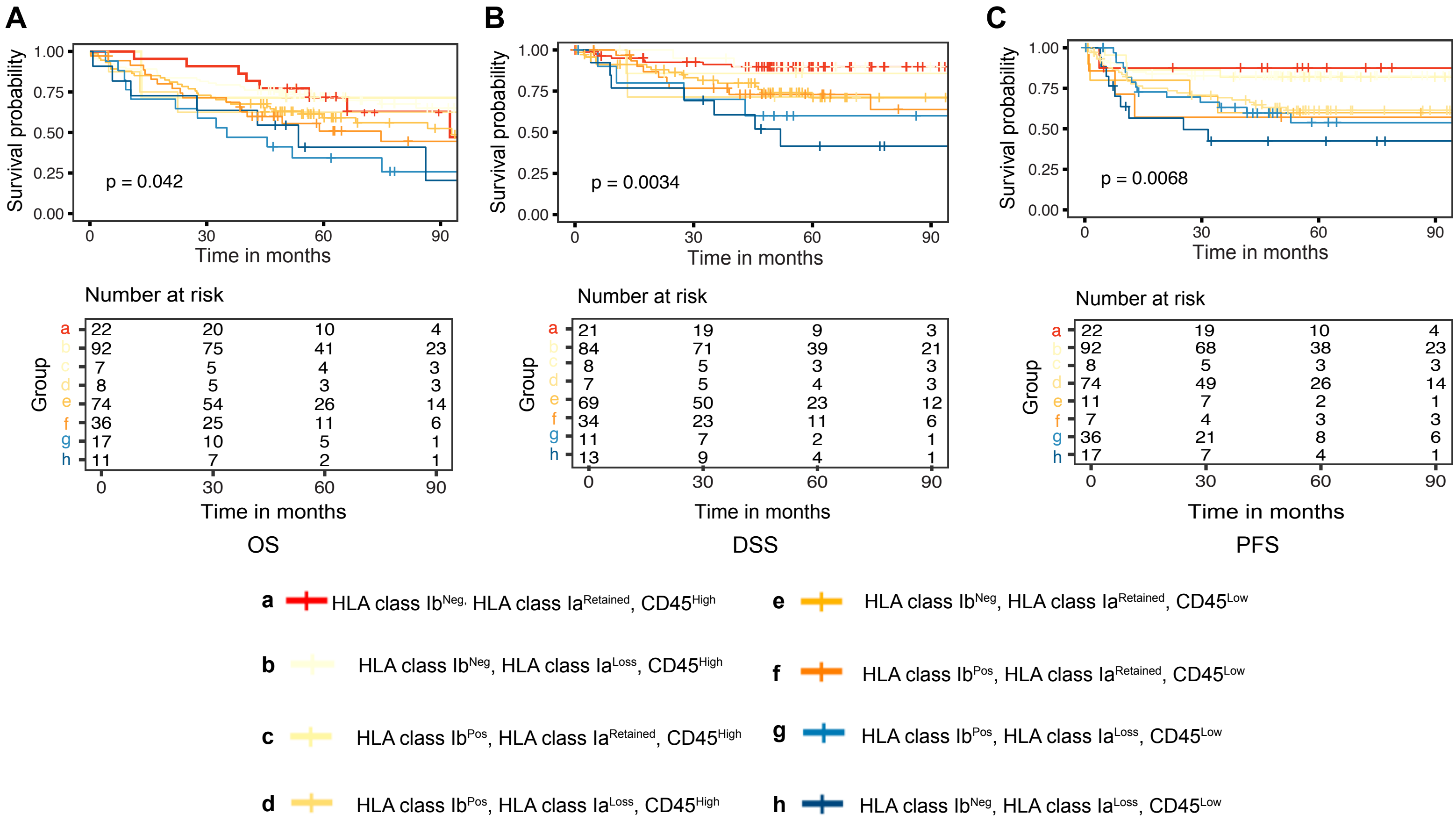
A
B
C
Survival probability
1.00
0.75
0.50
0.25
0.00
p = 0.042
p = 0.0034
p = 0.0068
0
30
60
90
Time in months
Number at risk
Group
a 22 20 10 4
b 92 75 41 23
c 7 5 4 3
d 8 5 3 3
e 74 54 26 14
f 36 25 11 6
g 17 10 5 1
h 11 7 2 1
OS
a 21 19 9 3
b 84 71 39 21
c 8 5 3 3
d 7 5 4 3
e 69 50 23 12
f 34 23 11 6
g 11 7 2 1
h 13 9 4 1
DSS
a 22 19 10 4
b 92 68 38 23
c 8 5 3 3
d 74 49 26 14
e 11 7 2 1
f 7 4 3 3
g 36 21 8 6
h 17 7 4 1
PFS
a HLA class Ib^Neg, HLA class Ia^Retained, CD45^High
b HLA class Ib^Neg, HLA class Ia^Loss, CD45^High
c HLA class Ib^Pos, HLA class Ia^Retained, CD45^High
d HLA class Ib^Pos, HLA class Ia^Loss, CD45^High
e HLA class Ib^Neg, HLA class Ia^Retained, CD45^Low
f HLA class Ib^Pos, HLA class Ia^Retained, CD45^Low
g HLA class Ib^Pos, HLA class Ia^Loss, CD45^Low
h HLA class Ib^Neg, HLA class Ia^Loss, CD45^Low
