# Supplementary material for: HLA class I expression and tumor immune infiltration together shape colon cancer immune contexture
Source: Neoplasia. 2026 Sep 2;81:101357. doi: 10.1016/j.neo.2026.101357 (PMC13570313; doi:10.1016/j.neo.2026.101357)
Supplement: Supplementary file 8 [file mmc9.pdf]

Characteristics	Number of cases (%)
**Patients**	280
**Median age (range), years**	70 (25-91)
**Sex**	
Male	154 (55.0)
Female	126 (45.0)
**Anatomical location**	
Right sided (ceceum, colon ascendens, flexura hepatica, colon transversum)	146 (52.2)
Left sided (flexura lienalis, colon descendens, colon sigmoideum, rectosigmoideum)	134 (47.8)
**Adjuvant treatment**	
Yes	89 (31.79)
No	191 (68.21)
**AJCC staging**	
I	41 (14.6)
II	101 (36.1)
III	85 (30.4)
IV	53 (18.9)
**MSI status**	
MSI-H	43 (19.0)
MSS	183 (81.0)
**CMS classification**	
CMS1	35 (12.5)
CMS2	63 (22.5)
CMS3	52 (18.6)
CMS4	61 (21.8)
Mixed	69 (24.6)
**ICR classification**	
Low	74 (26.4)
Medium	144 (51.4)
High	62 (22.2)
