# Supplementary material for: HLA class I expression and tumor immune infiltration together shape colon cancer immune contexture
Source: Neoplasia. 2026 Sep 2;81:101357. doi: 10.1016/j.neo.2026.101357 (PMC13570313; doi:10.1016/j.neo.2026.101357)
Supplement: Supplementary file 9 [file mmc8.docx]

**Supplementary Table Legend**

**Table S1**

Patients’ clinicopathological and genomic characteristics.

Data are presented as number of cases with corresponding percentages, unless otherwise specified. Detailed methods for molecular and immune classification are described in the Methods section. AJCC: American Joint Committee on Cancer; MSI: Microsatellite instability; MSS: Microsatellite stable; CMS: Consensus Molecular Subtypes; ICR: Immunologic Constant of Rejection.

**Supplementary Figure legends**

**Figure S1**

Study flow chart illustrating the cohort size, data completeness, and analysis strategy. All included cases were formalin-fixed paraffin-embedded (FFPE) primary colon tumors (n = 280). After immunohistochemistry (IHC) and quality control, cases were retained per marker and endpoint. For each survival outcome (overall survival, OS; disease-specific survival, DSS; progression-free survival, PFS), complete-case analyses were performed without imputation. Composite immune phenotypes were defined using the intersection of three markers: HLA class Ia, HLA class Ib (including HLA-E, HLA-F, and HLA-G), and intratumor epithelial CD45-positive immune cell density. Each analysis included only cases with complete data for all markers and the corresponding clinical endpoint. Further details regarding scoring and phenotype definitions were provided in the Methods.

**Figure S2**

Workflow of semi-automated image analysis for HLA class Ia expression in colon cancer. Representative IHC double-stained sections from two tumors (A and B) illustrate the sequential steps of tissue segmentation using the EMR8-5 antibody. Stromal tissue, blood vessels, and immune cells were immunohistochemically stained brown and HLA-I expression blue. Left: selection of total tissue area (red outline). Middle: selection of tumor epithelium after excluding stromal regions (red). Right: selection and quantification of HLA-I positive epithelial area (red). Percentages represent the proportion of HLA-I positive area within the selected epithelium. Scale bar is 200 *µ*m (A-B).

**Figure S3**

Representative immunohistochemical stainings of HLA class Ia and CD45 in colon cancer tissues.

Panels A–D show HLA class Ia staining (blue chromogen) and ECM (brown chromogen) with varying levels of membranous expression in tumor epithelium. (A) High expression of HLA class Ia; (B) Low expression of HLA class Ia; (C) Complete loss of HLA class Ia. Panels D–E show CD45 staining (brown chromogen), indicating leukocyte infiltration; tumor stroma structure stained with ECM (blue chromogen) (D) High infiltrated CD45-positive cells density; (E) Low infiltrated CD45-positive cells density. (F) Distribution of CD45-positive cell density (cells per mm²). Histogram with Freedman–Diaconis binning. Vertical reference lines mark Q1, median, mean, and Q3. The distribution is strongly right-skewed with a long high-value tail; (G) Empirical cumulative distribution function (ECDF) of CD45-positive cell density (cells per mm²). The vertical line marks the median (50th percentile), which was used as the primary cutoff for dichotomization because it is robust to skewness and outliers. Arrows in panels A–C indicate tumor epithelial regions with varying HLA class Ia expression levels. Scale bar is 200 *µ*m (A-E). Insets in D and E present higher magnification views of the boxed region, scale bar is 50 *µ*m.

**Figure S4**
Representative immunohistochemical stainings of non-classical HLA class I molecules in colon cancer tissues.

HLA-E (panel A) and HLA-G (panel B) were assessed using IHC double staining, where blue chromogen intensity indicates level of expression. HLA-F was evaluated by conventional IHC staining (panel C), where brown chromogen indicates level of expression. Each panel shows three expression categories: High (left), Low (middle), and Negative (right), based on staining intensity and distribution in tumor epithelium (see Methods). Scale bar is 400 *µ*m.

**Figure S5**

HLA-E expression across HLA class Ia expression groups.

Tumors were grouped by HLA class Ia expression (High, Low or Negative). HLA-E expression was categorized as High, Low, or Negative. A significant association was found between the two markers (*p* = 0.046, Chi-square test).

**Figure S6**

Stratified survival analyses of HLA-E, HLA-F and HLA-G expression in ICR-high tumors.

Kaplan–Meier plots showing progression-free survival (PFS; A, B, C) and disease-specific survival (DSS; D, E, F) in ICR-high tumors stratified by expression levels of HLA-E (A, D), HLA-F (B, E) and HLA-G (C–F). Groups were defined as Positive or Negative. Vertical lines indicate censor points. *P* values are two-sided.

**Figure S7**

Survival curves stratified by combined tumor expression of HLA class Ia, class Ib and CD45-positive cell density in colon cancer. (A–C) Overall survival (OS), disease-specific survival (DSS), and progression-free survival (PFS) stratified by all eight combinations of HLA class Ia (Retained or Loss), CD45-positive cell density (High or Low), and HLA class Ib (HLA-E/F/G) (Positive or Negative). OS: n=267; DSS: n=247; PFS: n=267. *P* values are from the global log-rank test across all groups. Vertical lines indicate censor points.
